# Characterization of a ripening-related transcription factor FcNAC1 from Fragaria chiloensis fruit

**DOI:** 10.1038/s41598-018-28226-y

**Published:** 2018-07-12

**Authors:** C. Carrasco-Orellana, Y. Stappung, A. Mendez-Yañez, A. C. Allan, R. V. Espley, B. J. Plunkett, M. A. Moya-Leon, R. Herrera

**Affiliations:** 1 Instituto de Ciencias Biológicas, Universidad de Talca, 2 Norte 685, Talca, Chile; 2 New Zealand Institute for Plant and Food Research Limited, Mt. Albert Research Centre, Auckland, 1025 New Zealand; 3 School of Biological Sciences, University of Auckland, Private Bag, 92019 Auckland, New Zealand

## Abstract

*Fragaria chiloensis* is a strawberry endemic from Chile with attractive white-pink fruit, pleasant aroma and taste. However, this fruit has a limited post-harvest period due to fast softening. Several transcription factors (TFs) are involved in the regulation of fruit ripening, and members of the NAC family have been implicated in cell wall remodeling. *FcNAC1* was isolated from *F*. *chiloensis* fruit, coding a protein of 332 amino acid residues and displaying a characteristic NAC domain at the N terminus. FcNAC1 protein showed nuclear localization. An increase in transcript level was observed during ripening. A sequence of 1488 bp of *FcNAC1* promoter was obtained. *In silico* analysis identified *cis* elements able to respond to some hormones and Secondary wall NAC binding elements (SNBE), and responding to auxin and ABA. A structural model of FcNAC1 provided evidence for interaction with DNA sequences containing SNBE, while a dual luciferase assay confirmed the transcriptional activation by FcNAC1 of the promoter of *FcPL*, a gene involved in cell wall remodeling in *F*. *chiloensis* fruit. The results suggest the participation of FcNAC1 during ripening development of strawberry fruit, by regulating pectin metabolism during softening.

## Introduction

The wild strawberry, *Fragaria chiloensis* L. Duch., is a native species from Chile, which is distributed from the arctic circle in the west of North America to the southernmost point of Chile and Argentina^1,2^. The species produces fruits, which follow a non-climacteric pattern, and are characterized by an attractive appearance and quality attributes such as taste and aroma, in addition to its high nutritional value imparted by elevated concentrations of minerals, vitamins and antioxidants^3^. Several attributes make the fruit of *Fragaria chiloensis* an attractive agronomic resource, but the accelerated fruit softening and a short flowering reproductive period are negative aspects affecting production^4,5^.

The fruit ripening process comprises a highly coordinated, irreversible and genetically programmed event which involves many biochemical, physiological and organoleptic modifications that lead to the development of a soft and edible ripe fruit with ideal quality features. This is concomitant with various changes such as chlorophyll degradation, carotenoid or anthocyanin biosynthesis, increased respiration, essential oils, flavor and aroma components, increased activity of cell wall-degrading enzymes, and transient increases in hormonal production that take place during fruit ripening^6^. During ripening of fleshy fruit important modifications in the cell wall structure take place related with wall components whose solubility percentage increases, polymer length decrease, and linkages between many kinds of polymers are modified to produce a decrease in fruit firmness^7–9^.

NAC genes are a transcription factor family (TF), which have been found to play important roles in plant development and environmental responses. Members of this family have been reported to be involved in the ripening and softening of fleshy fruits such as citrus^10^, banana^11^, tomato^12^, and peach^13^. The acronym of NAC comes from the first three genes described, which contain the domain: NAM (No Apical Meristem) from petunia^14^, ATAF1 and ATAF2 (GenBank accession numbers X74755 and X74756) and CUC (Cup-Shape Cotyledon) from *Arabidopsis thaliana*^15^. NAM has been associated in determining positions of meristems and primordia in petunia^14^. ATAF1 and ATAF2 are negative regulators of defense response against pathogens in Arabidopsis^16,17^, while CUC genes are involved in shoot apical meristem (SAM) formation and cotyledon separation in the embryogenesis^18^. These TFs have been identified as large multi gene families^19^: 117 genes have been described in Arabidopsis, 151 in rice, 79 in grape, 26 in citrus, 163 in poplar, 152 in soybean and 152 in tobacco^20–23^. These TFs share a NAC domain consisting of 130–150 amino acids at their N-terminus that allows for their interaction with DNA, which is divided into five subdomains, A–E, from N-terminus to C-terminus respectively^24^. The protein also contains a transcriptional regulatory (TR) domain at the C-terminal region, and some NAC TFs have a transmembrane domain within the TR domain^25^. Phylogenetic analyses of NACs from nine plant species revealed that these TFs are divided into 21 subfamilies, some of which are specific to either monocots or eudicots^26^.

To date there are some reports involving members of the NAC family in processes related to ripening of fleshy fruit. For example, in tomato (*Solanum lycopersicum*) SlNAC4 shows high transcript accumulation in sepals and the early stages of ripening. RNAi assays of SlNAC4 results in delays to the ripening process of tomato fruit, by reducing transcript levels of genes related to the ethylene pathway and a decrease in carotenoid content^12^. Other reports have shown that the overexpression of *SlNAC1* results in a reduction of carotenoids by altering carotenoid pathway flux and decreasing ethylene synthesis mediated mainly by reduced expression of ethylene biosynthetic genes, thus leading to yellow or orange mature fruits^27^. In banana (*Musa acuminata*) six NAC genes (*MaNAC1*-*MaNAC6*) have been described to show a differential expression profile between skin and pulp, in which MaNAC1 and MaNAC2 play a direct role in fruit ripening^11^. More recently, a NAC gene from kiwifruit has been associated with monoterpene production, and it has been proposed to be crucial in promoting the synthesis of aroma during ripening^28^. Information about NAC transcription factors related to cell wall remodeling in fleshy fruits has not been widely described. The genes encoding the suite of enzymes required promoting cell wall remodeling needs to be coordinately regulated in a temporal and spatial manner. In this sense, the activation of secondary wall biosynthetic genes is modulated by a transcriptional network, including secondary wall NAC (SWN) master switches and their downstream transcription factors^29^.

The role of NAC TFs in the ripening and softening process of Chilean strawberry is still unknown. A partial fragment of a NAC gene was identified as differentially expressed in a suppressive substractive hybridization (SSH) library prepared from Chilean strawberry fruit^30^. Using rapid amplification of cDNA ends (RACE) the full-length coding sequence was obtained. This gene, termed *FcNAC1* was characterized to elucidate its participation during the ripening of the Chilean strawberry fruit. This characterization included the analysis of its sequence and the confirmation of a functional DNA binding domain, its nuclear localization, and attempts to explain its transcriptional regulatory effect on genes related to cell wall remodeling associated with fruit softening. To complete the study the role of hormones that participate in ripening of strawberry fruit on the transcriptional regulation of FcNAC1 was analyzed.

## Results

### Cloning the Full-length of FcNAC1 and Sequence Analysis

**Figure 1:**
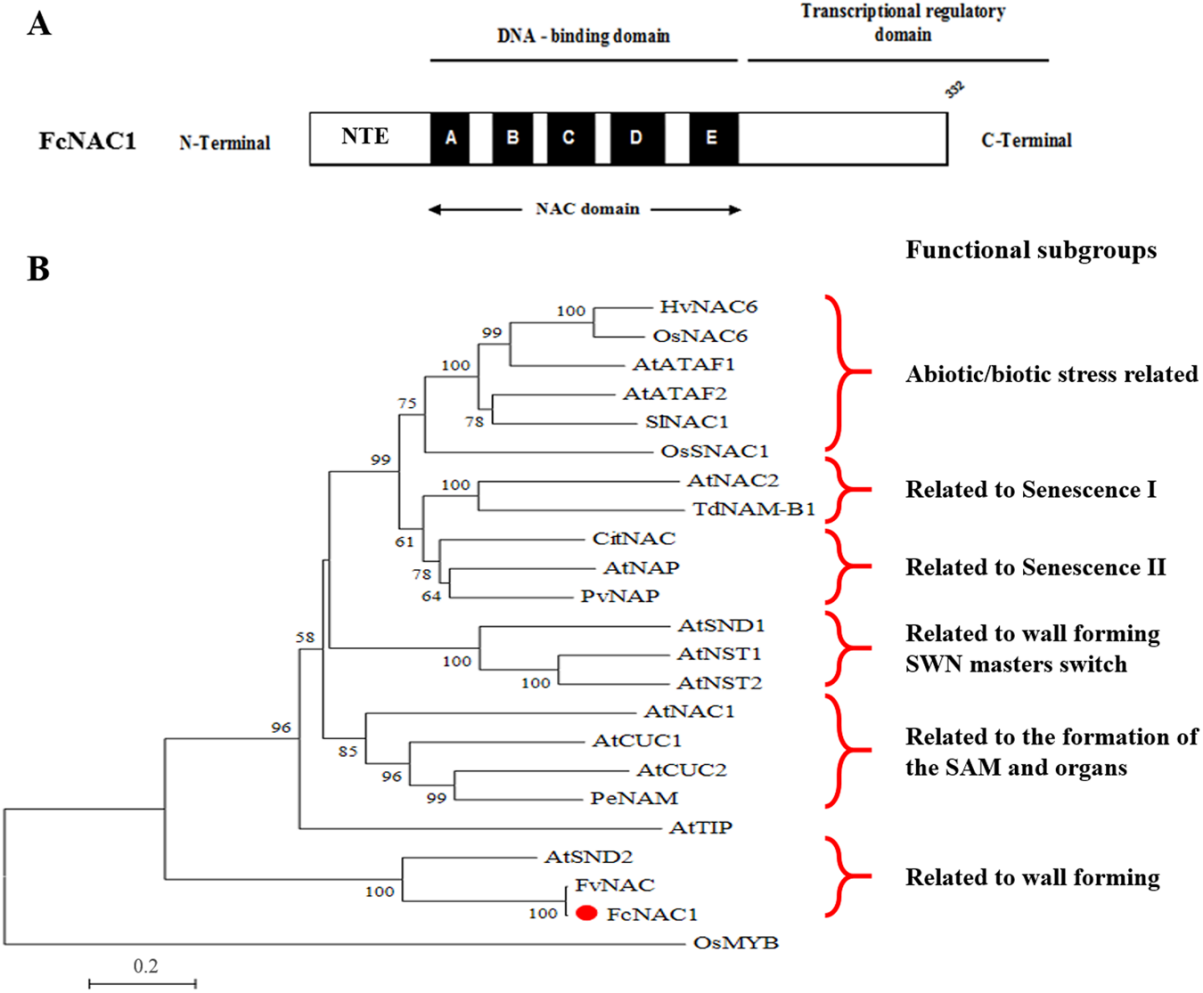
Schematic representation of FcNAC1 protein and phylogenetic analysis with NAC sequences. (**A**) Schematic representation of the primary structure of FcNAC1 showing the NTE (N terminal extension), the DNA binding domain (NAC domain) divided into five subdomains (A to E), and the transcriptional regulatory domain, as described in Puranik *et al*.^24^. (**B**) Phylogenetic analysis where FcNAC1 groups with members of the NAC family related to wall formation, close to AtSND2. We used amino acid sequences of NAC members with described function: AtNAC2 (AB049071.1), AtNAP (NM_105616.4), AtCUC1 (EU550396.1), AtCUC2 (AB002560), HvNAC6 (AM500854.1), AtNAC1 (NP_175997), TdNAM-B1 (KF541318), AtNST1 (NM_130243), AtSND1 (EF101892), AtSND2 (NC_003075.7), AtNST2 (NP_191750), OsNAC6 (AB028185.1), PeNAM (X92205), PvNAP (XP_007158644), SlNAC1 (NM_001247553.3), OsSNAC1 (KM265360.1), and AtTIP (NP_197847). The transcription factor OsMYB (CAA72218) was used as outlier. The phylogenetic tree was performed with MEGA5 software employing the neighbor joining method, with 1000 replicates.

Starting from a partial fragment (939 bp) of a putative NAC gene^30^ the full-length sequence of FcNAC1 was cloned from ripe strawberry fruit RNA samples using 3′ RACE method, employing FcNAC1-RACE1 and FcNAC1-RACE2 primers (Table 1). After performing the two RACE reactions, two fragments of 373 bp for FcNAC1-RACE1 and 461 bp for FcNAC1-RACE2 were obtained. The combined *FcNAC1* sequence (GenBank accession number AKC96459.1) contained an ORF of 999 bp and codes for a deduced polypeptide sequence of 332 amino acid residues with a theoretical molecular mass of 37.3 kDa and an isoelectric point of 7.0. FcNAC1 shares a 99.4% amino acid identity with FvNAC and 62.6% with SND2 (Supplementary Table 1). FcNAC1 protein sequence contains a highly conserved region towards the N-terminal, corresponding to the NAC domain which is divided into five sub-domains, A to E (Fig. 1A). The sub-domain C contains the nuclear localization signal (Supplementary Fig. 1). The protein contains an extension at the N-terminal (NTE). The transcriptional regulatory domain identified at the C-terminal displays low similarity among the sequences. A transmembrane domain was not predicted in the FcNAC1 protein.

**Table 1:** Nucleotide sequence of the primers (5′ → 3′) used in this study.

Name	Forward primer sequence	Reverse primer sequence
FcNAC1-RACE1	AGGAGGCCAGAATAGGGAAA	
FcNAC1-RACE1	TTCCTCCAAAGAAGGCTGGT	
FcNAC1-Realtime	TGGTATGAGCGGCCTCAG	GCTGCCCTCTCTTCTTCCTC
FcGAPDH1	TCCATCACTGCCACCCAGAAGACTG	AGCAGGCAGAACCTTTCCGACAG
FcNAC1-Full-length	CACCATGACATGGCACTCAGATGAGG	GTTTCCTTTGCTTGTATCTGCA
FcNAC1-GW1		CCTCTCTTCTTCCTCTTCCTCATCTG
FcNAC1-GW2		GCAGAGGAGGGAGTTATTGACTGAAC
FcNAC1-GW3		CTATAGGGTGGCCACAAGACG
FcNAC1-GW4		GAGTGGATGAAGCTTTCTGGTATC

### Phylogenetic analysis of FcNAC1 protein

**Figure 2:**
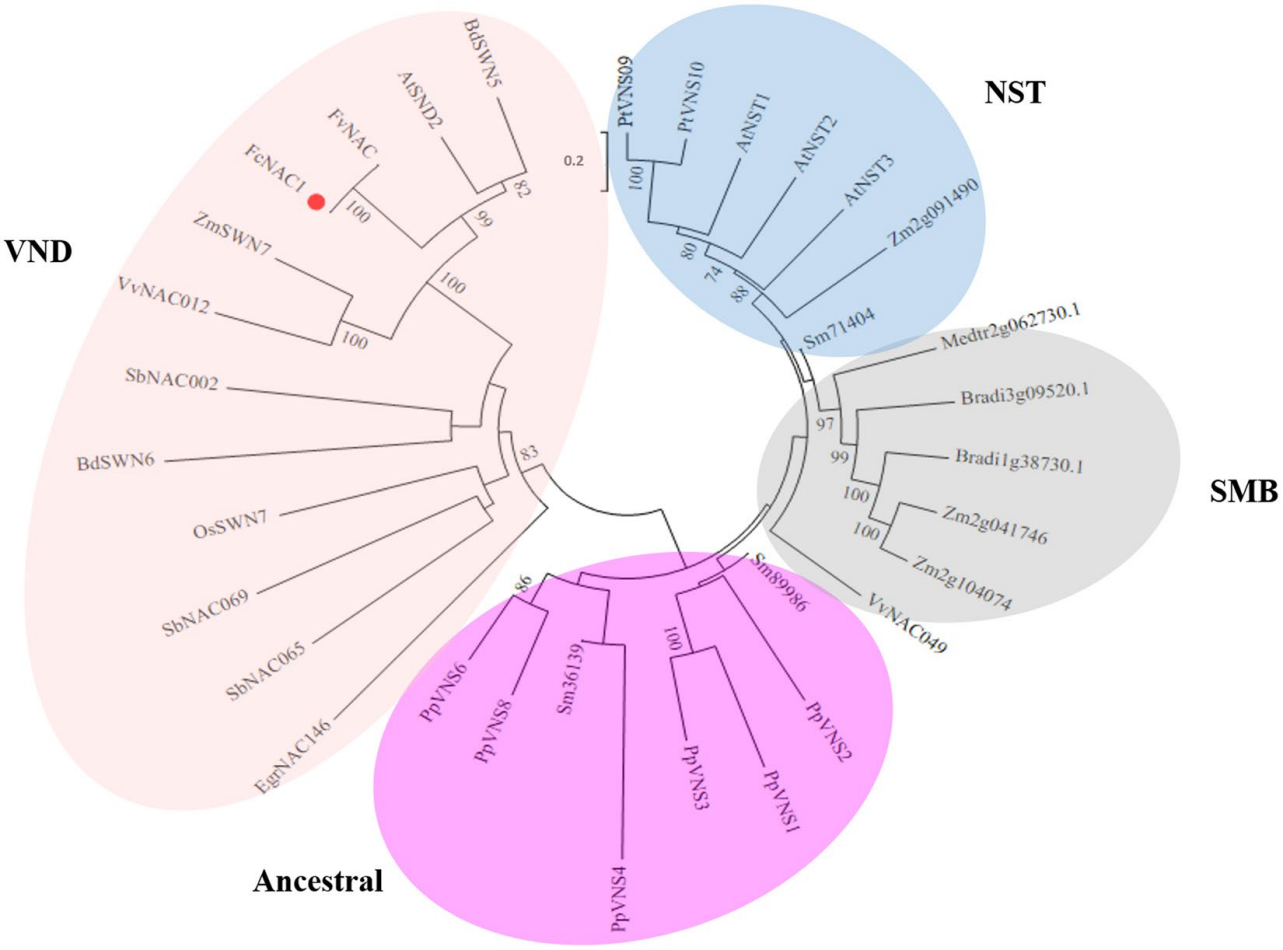
Phylogenetic analysis of FcNAC1 with VNS (*V*ND, *N*ST and *S*MB) proteins as Master Regulators of cell wall formation. In the analysis, members of the NAC family with function related to cell wall remodeling were included. Representative genes of NST, SMB, VND and ancestral groups were considered for the analysis^32^. The phylogenetic tree was performed with MEGA5 software employing the neighbor joining method, with 1000 replicates.

A phylogenetic analysis was performed to reproduce the different groups with known function that have been previously reported^31^. The analysis included 21 NAC protein sequences including representatives from Arabidopsis, tomato, barley, rice, citrus, petunia and bean with known function, and one homologous sequence from woodland strawberry (*Fragaria vesca*). FcNAC1 protein was grouped into the cluster related to cell wall related NACs, near to FvNAC and SND2 (Fig. 1B). Additionally, a phylogenetic tree including 33 other recently reported proteins^32^ was built, and FcNAC1 grouped in the Vascular-related NAC Domain (VND) cluster (Fig. 2). FcNAC1 was clustered in the same branch with SND2, a member of the NAC family related to cell wall formation.

### Subcellular localization of FcNAC1 protein

**Figure 3:**
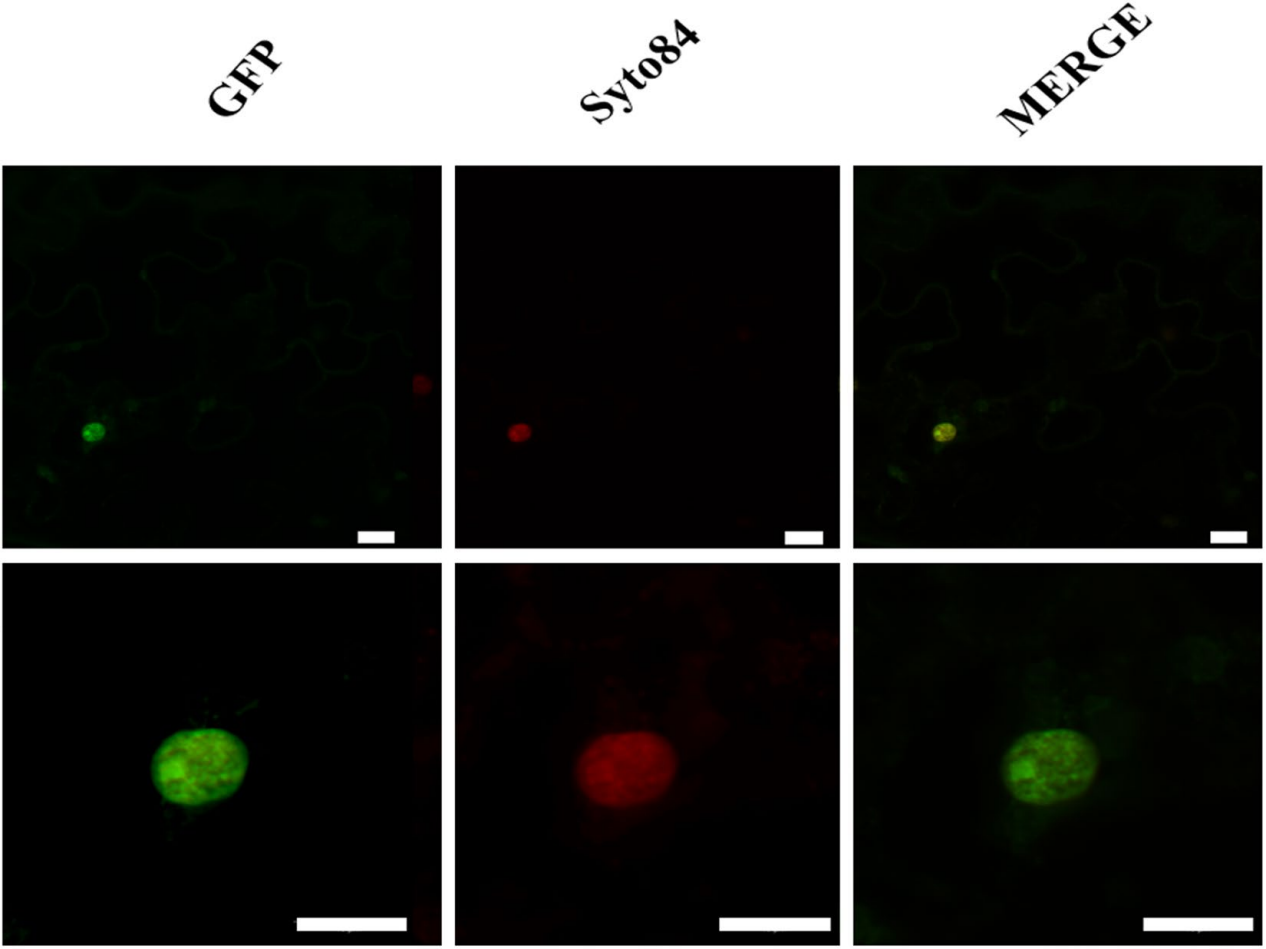
*FcNAC1* subcellular localization of the transcription factor by transient transformation of tobacco leaves. Co-localization assay of FcNAC1 protein fused to GFP in vector pK7WGF2.0. *Agrobacterium tumefaciens* LV300 strains were used to transiently transform young leaves from tobacco plants (6-week-old). Syto84 staining was used to locate the nucleus. GFP: Green Fluorescent Protein, Syto84: nuclear staining, MERGE: superposition of GFP and Syto84 images. The white bar indicates 10 µm.

As a transcription factor, FcNAC1 should be localized in the nucleus. Its nuclear localization was predicted using the web server NLS Mapper (http://nls-mapper.iab.keio.ac.jp/cgi-bin/NLS_Mapper_form.cgi) (Supplementary Fig. 2). In order to demonstrate its *in vivo* subcellular localization the coding sequence was fused to GFP protein sequence and cloned into pK7FWG2.0 vector under the control of 35 S promoter. The plasmid was transfected into Agrobacterium (LBA4404) and then tobacco leaves were agro-infiltrated. After 3 days the fluorescence from FcNAC-GFP fusion protein was measured. A strong fluorescence signal was detected in the nucleus (Fig. 3), which co-localized with the fluorescence of the nuclear stain SyTo^®^ 84, confirming the nuclear localization of FcNAC1.

### FcNAC1 transcript levels in developing strawberry fruit and other tissues

**Figure 4:**
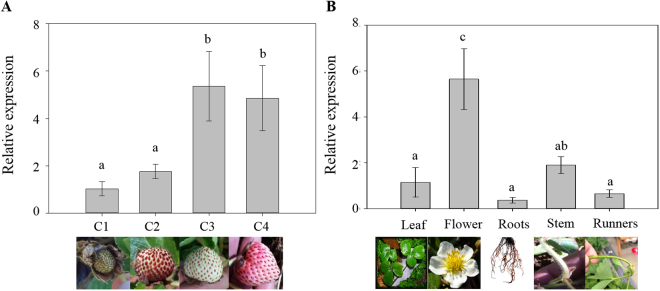
Levels of *FcNAC1* transcripts in different *F*. *chiloensis* tissues. Quantification of transcripts was performed by qRT-PCR employing specific primers: (**A**) during development and ripening of *Fragaria chiloensis* fruit (C1, C2, C3 and C4); (**B**) different plant tissues (leaves, flowers, roots, stem and runners). RNA extractions from the samples were performed using the CTAB method. *FcGAPDH* was used as internal calibrator gene. Different letters indicate statistical difference between fruit stages (**A**) or plant tissues (**B**). Significant differences were determined at *p* ≤ 0.05 (LSD Fisher test). In order to facilitate the comparison of transcripts level, C1 fruit stage in A, and leaf in B were normalized to 1. All pictures were taken by one of the authors.

*FcNAC1* displays a differential expression pattern during fruit development in *F*. *chiloensis* (Fig. 4). *FcNAC1* showed low transcripts level at C1 and C2 stages, which correspond to unripe fruit stages. An increment in the transcriptional level occurs at the C3 stage, maintaining a high transcript level at C4 stage (Fig. 4A). Other tissues were tested to identify the specific expression of *FcNAC1*. Gene expression analysis showed that *FcNAC1* transcript abundance was high in flowers when compared with the other vegetative tissues. Similar low transcript levels were detected in roots, runners and leaves (Fig. 4B).

### Changes in the expression of FcNAC1 by hormones

**Figure 5:**
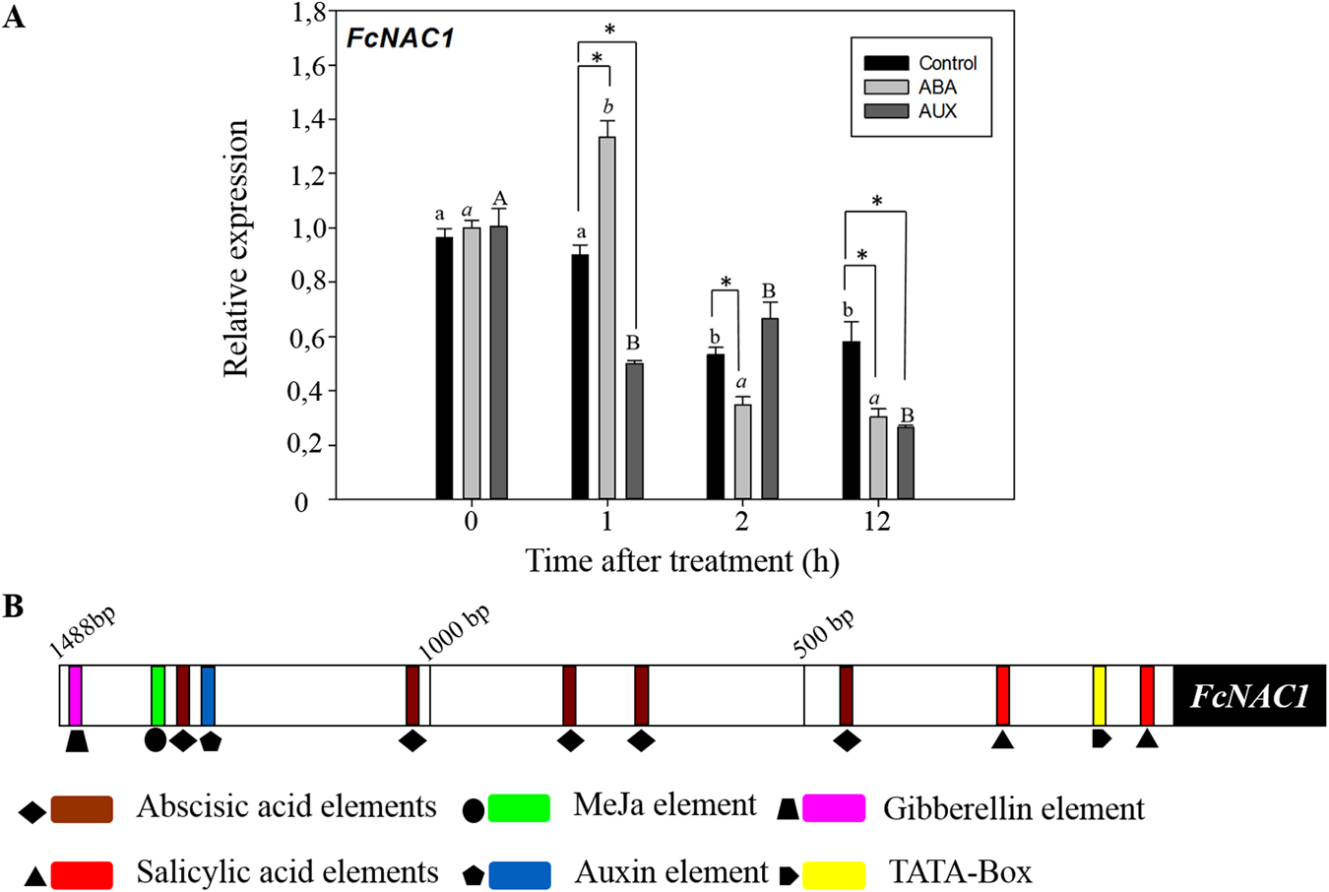
Effect of hormones on the transcription of *FcNAC1*. (**A**) Expression changes of *FcNAC1* in response to ABA and auxins. *F*. *chiloensis* fruit at the C2 stage was subjected to hormonal treatments (1 mM ABA and 1 mM ANA) or immersed in buffer (control). Samples were collected just after treatment (10 min), and after 1, 2 and 12 h, and stored at −80 for subsequent RNA extractions by the CTAB method. Expression analyses were performed through qRT-PCR. For the same treatment, different letters indicate significant differences during the observation period. Significant differences were determined at *p* ≤ 0.05 (LSD Fisher test). (**B**) Analysis of *FcNAC1* promoter. By means of the genome Walker technique, a sequence of 1488 bp corresponding to the promoter sequence of FcNAC1 was obtained and *in silico* analyzed with PlantCARE database (http://bioinformatics.psb.ugent.be/webtools/plantcare/html/). Different putative cis-elements related to several hormonal responses were identified along the sequence.

In order to test the hormonal effect on the transcription of *FcNAC1*, fruit at the C2 stage were treated with ABA or synthetic auxin (NAA) (Fig. 5A). The relative expression level of *FcNAC1* in untreated C2 fruit (control) displayed a decreasing expression pattern during the observation period (12 h at room temperature). In response to ABA, a rapid accumulation of *FcNAC1* transcripts was observed after 1 h of treatment, and then a significant reduction was observed after 2 and 12 h. In contrast, a reduction in the expression level of *FcNAC1* transcripts was observed after 1 h of auxins treatment, and maintained during the following 12 h. These results indicate that FcNAC1 displays a different expression response to ABA and auxins.

### Analysis of FcNAC1 promoter sequence

In order to understand the transcriptional regulation of *FcNAC1*, its promoter sequence was isolated. A 1488 bp sequence was obtained from genomic *F*. *chiloensis* DNA. An *in silico* analysis was carried out to search for putative *cis*-regulatory elements related to hormonal response. The promoter of FcNAC1 contains five ABA-related sequences, in addition to sequences related to auxins, gibberellins, methyl jasmonate and salicylic acid responses (Fig. 5B). The *cis*-regulatory elements identified in the promoter of FcNAC1 might explain their differential hormonal response. Putative *cis*-regulatory elements for NAC binding were also identified in the promoter (Supplementary Fig. 3).

### Functional analysis of FcNAC1 gene and promoter sequences in transiently transformed tobacco

**Figure 6:**
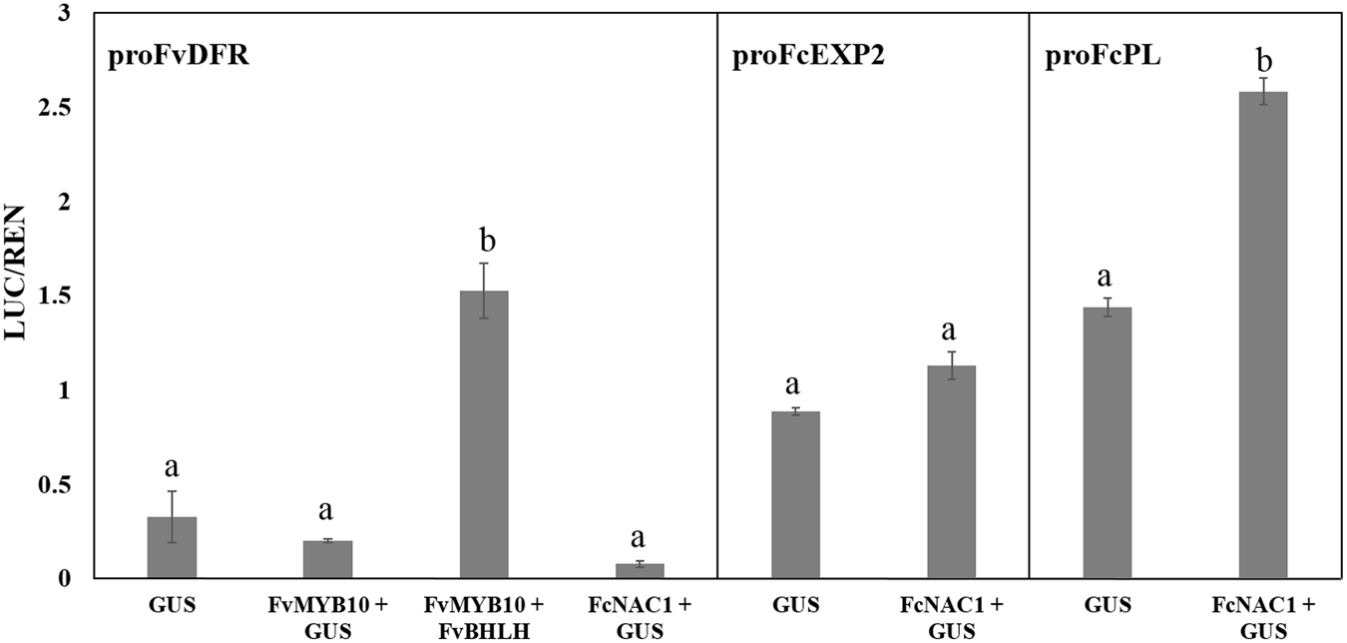
Functional assay of *FcNAC1* gene by transient transformation of tobacco leaves. The ratio of LUC/REN activities obtained after Dual Luciferase interaction assay of *FcNAC1* with the promoter sequences of *FvDFR* (Dihydroflavonol reductase, 1560 bp) (XP_004308377), *FcEXP2* (Expansin 2, GenBank KC527027, 847 bp) and *FcPL* (Pectate lyase, GenBank KC527025, 1038 bp) in transiently transfected *N*. *benthamiana* leaves are shown. *GUS* represents the plasmid 35SPro-*GUS* (negative control). *FvMYB10* (GenBank EU155163.1) and *FvBHLH* (Basic helix loop helix XP_004308377; 1932 bp) were used as positive controls of the technique. Bars represent the bioluminescent measure of 4 replicates ± standard deviation. Different letters indicate statistical differences (*p* ≤ 0.05) compared to respective *GUS* control.

For functional analysis independent genomic constructs were built for *FcNAC1* (35 S:*FcNAC1-GFP*) and for the promoters of two cell wall degrading genes (*FcExp2* and *FcPL)* (promoter:*LUC*) which have been reported to be important for *F*. *chiloensis* softening^33^. These constructs were cloned and transfected into Agrobacterium (GV3101), then agro-infiltrated into *N*. *benthamiana* leaves for transient expression^34^. The expression of FcNAC1 protein did not change the Luc/Ren activity ratio in the case of the promoter of *FcExp2*, however a significant increase was observed in the activity of the promoter of *FcPL* (Fig. 6). This indicated that the FcNAC1 protein is able to activate the promoter of *FcPL*. As a positive control FvMYB10 and FvbHLH, increased the activity of the FvDFR promoter, a gene described to be transcriptionally regulated by these two transcription factors^34^. There were no significant differences in the transactivation activity of *FvDFR* promoter sequence by FcNAC1.

### Structural modeling of FcNAC1’s NAC domain

**Figure 7:**
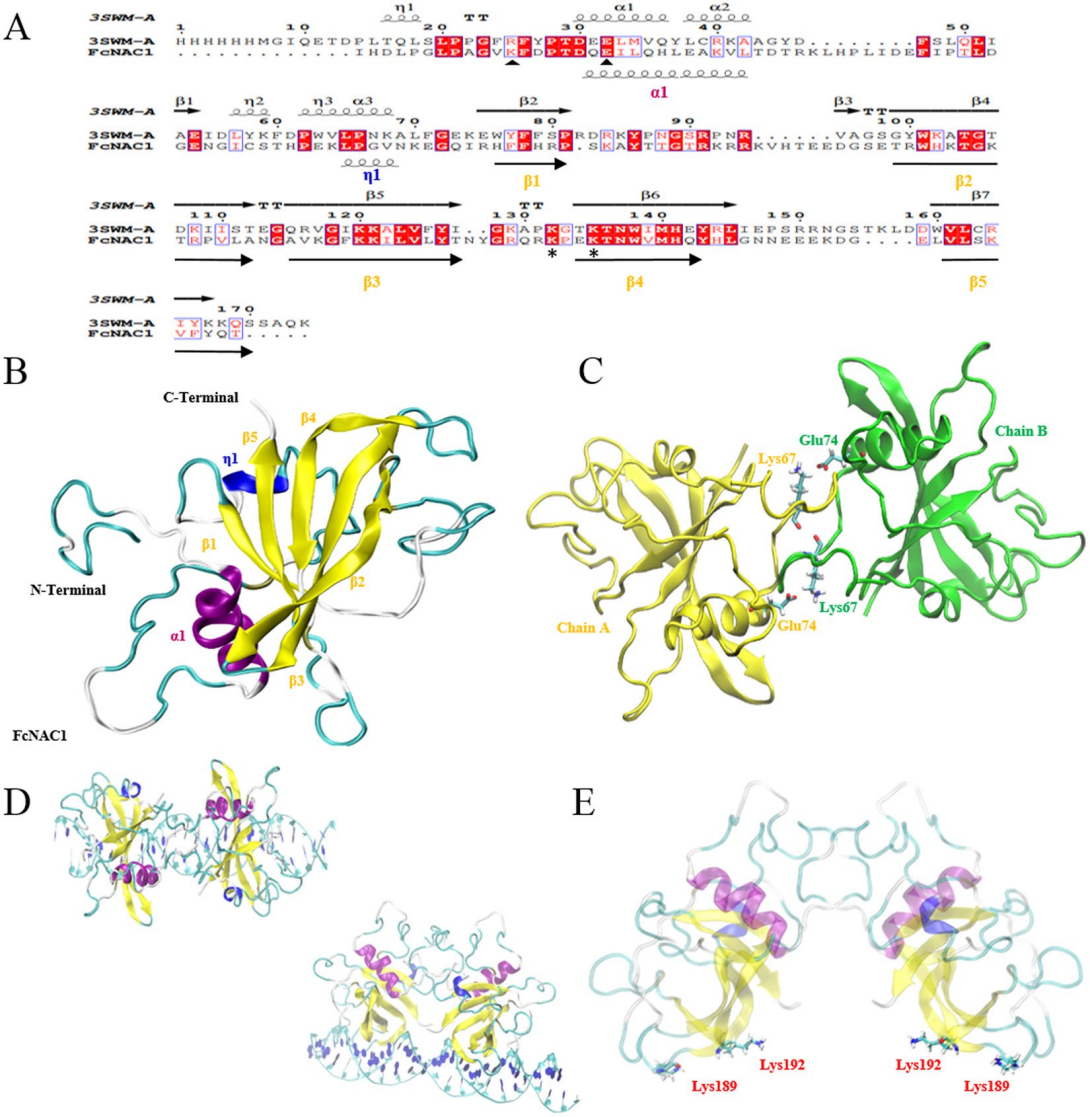
Homology modeling of the NAC domain present in FcNAC1 protein. (**A**) The alignment between the template (3SWM chain A) and FcNAC1 NAC domain, including structural information. Spirals and arrows at the top of the sequences represent the secondary structure of the template, while those at the botton correspond to predicted secondary structure of FcNAC1. Triangles indicate the important residues for the dimerization process and asterisk indicate conserved residues for DNA interaction. (**B**) Homology modeling of FcNAC1 NAC domain using MODELLER software. Fifty models were built and the best was subjected to energy minimization and molecular dynamics with the purpose of relaxing the structure and minimizing steric impediments. It is possible to appreciate that the cluster of β sheets is flanked on both sides by the α-helix and the α-helix 3_10_. α-helices were labeled as α, and β-sheets as β. (**C**) Homodimer structure of FcNAC1 NAC domain showing the potential residues involved in the salt/bridge interaction. (**D**) Protein-DNA positioning between the homodimer of FcNAC1 and the DNA sequence co- crystallized with the template 3SWM (5′- GTCTTGCGTGTTGGAACACGCAACAG -3′). (**E**) Visualization of the homodimer structure of FcNAC1 NAC domain highlighting the positively charged residues involved in the protein-DNA interaction.

The 3D monomer structure of FcNAC1 NAC domain was built using the chain A of crystallographic structure of ANAC019 from *Arabidopsis thaliana* (PDB code: 3SWM) as template. The pairwise alignment of the NAC domain from FcNAC1 (from residue 56 to 222) predicted with SMART software (http://smart.embl-heidelberg.de/) (167 residues in total) against chain A of the template shares 28.5% identity (Fig. 7A). In terms of geometric stability, 2 nanoseconds of Molecular Dynamics Simulation (MDS) were performed and the protein model showed a RMSD of ~1.5 Å (Supplementary Fig. 4). The structural model was validated through PROCHECK, showing that 82.4% of the residues were located in the most favoured regions with no poorly modeled residues identified (Table 2). In addition, ProSA analysis confirmed the good quality of the model (Z-score: −4.64) (Supplementary Fig. 5). The final structural conformation of FcNAC1 NAC domain forms a twisted anti-parallel β-sheet, which is composed of one α-helix, one α-helix 3_10_, five β sheets and eight loops. It can be observed that this β-sheet is packed between an N-terminal α- helix and a short α-helix 3_10_ on the other side (Fig. 7B).

**Table 2:** Validation of FcNAC1 NAC domain structure by PROCHECK program.

	Core (%)^**a**^	Allow (%)^**b**^	Gener (%)^**c**^	Disall (%)^**d**^
FcNAC1-NAC domain	82.4	16.8	0.8	0

^a^Most favorable regions.

^b^Additional allowed regions.

^c^Generously allowed regions.

^d^Disallowed regions.

The FcNAC1 homodimer structure using both chains of the template, A and B, was built (Fig. 7C). The physical interaction of both monomers involved the conserved N-terminal sequence. The dimerization interaction involved two prominent salt bridges between Lys67 and Glu74 from adjacent chains. On the other hand, the interaction of the homodimer with DNA was also tested, showing that β-sheets β3 and β4 are involved in protein-DNA interaction, allowing the recognition of major DNA grooves (Fig. 7D). Additionally, the presence of positively charged residues (Lys189 and Lys192) in this domain, allows the interaction of the DNA sequence and FcNAC1 transcription factor (Fig. 7E).

## Discussion

The characterization of the *F*. *chiloensis* NAC transcription factor, named *FcNAC1*, revealed a protein sharing a highly conserved NAC domain and a variable C-terminal region that can act as a domain of transcriptional activation. The domains identified in FcNAC1 were initially described in *A*. *thaliana* and *Oryza sativa*^35^. Multiple alignments showed that FcNAC1 shares high similarity with sub-domains B, C and D from FvNAC and SND2 proteins.

A nuclear localization signal was predicted which was confirmed by the transient transformation into tobacco cells of FcNAC1 fused to a reporter gene. Other members of this NAC family also contain the same nuclear localization: a NAC from *S*. *lycopersicum* was shown to be located in the nucleus of onion epidermal cells^36^, as was also the case for AtNAC2^36^. MtNST1, described as a secondary cell wall master switch, was also identified in the nucleus of epidermal tobacco cells^37^. The tomato SlNAC3 has been localized in the nucleus of onion epidermal cells and reported to respond to environmental and endogenous stimuli and is likely to function during the response of plants to salt and drought stresses^38^.

The different NAC protein sequences characterized to date can be grouped into six functional sub-groups through phylogenetic analysis, which confirms the groups described previously^31,32^. In this case, FcNAC1 grouped with SND2, which has been related to cell wall formation^39^. The overexpression of *SND2* in *Arabidopsis* up-regulates the biosynthesis pathways of genes associated to cellulose, xylan, mannan, pectins and lignin polymerization, playing a crucial role in the secondary cell wall transcriptional network^40^. Recently a new classification has grouped NAC genes into three groups^32^. *FcNAC1* grouped in the VND cluster, as well as SND2, which is involved in cell wall biosynthesis. *FcNAC1* could be involved in similar functions, contributing to the fruit softening process.

The ripening of *F*. *chiloensis* fruit, and in particular its softening process, has been studied and several enzymes related to cell wall assembly/disassembly have been shown to increase during ripening. The transcript abundance of *FcEXP2* (*Expansin 2*), *FcPL* (*Pectate lyase*), *FcPG* (*Polygalacturonase*)^5^ and *FcXTH1* (*Xyloglucan transglycosylase/hydrolase*) all increased during ripening development of *F*. *chiloensis* fruit, as well as their activity. In addition, it has been reported that fruit firmness dramatically falls during the transition from C2 to C3 stage^41^. In accordance with this profile, *FcNAC1* transcripts level increase in the same developmental period, remaining at a high level until the end of fruit ripening. Kang *et al*.^42^ reported transcriptomic analysis during development of *Fragaria vesca* fruit, starting from flowering. Interestingly, *FvNAC* gene, with close homology to *FcNAC1*, showed a high accumulation of transcripts after nine days post anthesis. This indicated that the expression pattern is similar in *F*. *chiloensis* and *F*. *vesca*. Coincidently, a peak of transcript accumulation was observed in flowers of both species. In *F*. *vesca*, transcripts coding for *EG*, *Exp2*, *PG*, *PL* and *XTH* were also identified as differentially expressed during fruit development^41^. Moreover, the analysis of the promoter sequences of these genes in *F*. *vesca* contains cis-elements that are recognized by NAC members. Therefore, the involvement of *FcNAC1* in the regulation of genes related to cell wall remodeling might be expected.

In the commercial strawberry *Fragaria x ananassa*, the concentration of auxins decays during development of the fruit, while the concentration of abscisic acid increases^43^. The literature describes that abscisic acid (ABA) plays a crucial role in the regulation of strawberry fruit ripening and exogenous applications of this hormone promotes the ripening of this berry^44^. There is information about members of the NAC family and its relationship with different types of stresses, where it has been possible to show the different responses by NAC members from various species such as *S*. *lycopersicum*, *A*. *thaliana*, *Oryza sativa*, *S*. *tuberosum*, *Triticum aestivum*, *Brassica napus*, *Zea mays*, *sugarcane*, *Petunia hybrida and Citrus sinensis* to diverse kinds of stimuli (heat, cold, salt, water, wounding, insect/pathogen, methyl jasmonate and abscisic acid) producing variations in the transcription profiles of these NACs members^45^. Information is available on the specific modulation of diverse NAC transcription factor members by ABA; *ANAC019*, *ANAC055*, *ANAC072*, *RD26*, *RD20*, *ZmSNAC1*, *ANAC2*, *SNAC2*, *OsNAC5*, *OsNAC10*, *DgNAC1* and *CarNAC3* respond to ABA and, in some cases, ABA signaling^46^. In agreement with this, cis-elements related to hormonal response were identified in the promoter of *FcNAC1* suggesting the participation of hormones in its transcriptional regulation. ABA and auxins were able to modulate the accumulation of *FcNAC1* transcripts as it has been previously reported in *S*. *lycopersicum*^45^. An increase in the accumulation of transcript of *FcNAC1* by ABA and a decrease in the transcripts level after treatment with auxins were observed in *F*. *chiloensis* fruit.

The subgroup of NAC TFs related to cell wall biosynthesis (SWN, Secondary wall NAC domain) are able to promote the expression of diverse transcription factors, such as *SND2*, *SND3*, *MYB46*, *MYB103*, *MYB85*, *MYB52*, *MYB54*, *MYB69*, *MYB43*, *MYB20*, and *KNAT7*, which led to the proposal that a transcriptional network including SWNs and their downstream targets is involved in the regulation of secondary wall biosynthesis^46^. This activation can be carried out by binding an imperfect palindromic 19-bp consensus present in the promoter sequences and designated as a secondary wall NAC binding element (SNBE) (T/A)NN(C/T) (T/C/G)TNNNNNNNA(A/C)GN(A/C/T) (A/T). Studies using the reporter gene *GUS* showed that SWNs bind to SNBE sequences in the promoters of their direct target genes, and thereby activate their expression in the secondary wall-forming cells^47^. The same SNBE elements have been identified in the promoter sequence of *FcNAC1* containing the conserved CGT [A/G] core sequence that is present in the SNBEs sequences. The interaction between NAC TFs and these motifs have been reported using EMSA experiments, demonstrating that NAC proteins bind DNA as dimers^48^.

The dual luciferase experiment performed in this work shows the transactivation of a cell wall remodeling gene by a NAC TF of strawberry. NAC TFs are able to modulate the expression of genes acting during fruit ripening, such as those involved in color development^13^ or aroma formation^28^. FcNAC1 interacts with the promoter sequence of *FcPL*, increasing the expression of genes controlled by this promoter. However, the response was not observed with the promoter of *FcEXP2* or the control promoter, *FvDFR*. This evidence allowed us to hypothesize a possible role of *FcNAC1* in the transcriptional regulation of genes related to pectin metabolism, and in this way, contributing to the process of cell wall remodeling during development and softening of the *F*. *chiloensis* fruits.

The 3D structure of the NAC domain present in FcNAC1 was obtained through homology modeling. The homodimeric structure is stabilized by salt-bridges between Lys67 and Glu74 from adjacent FcNAC1 chains. In the literature it has been described that dimerization is supported by Arg25 and Glu32 in the case of ANAC019^49^. We hypothesized that Lys could be fulfilling the role of Arg in the dimerization process. On the other hand, NAC, WRKY and GCM TF families share a central β-sheet domain with similar topology, which is responsible for mediating the interaction with DNA^49^. The FcNAC1 model showed a motif between β-sheets β3 and β4, which may participate in protein-DNA interaction, and in agreement, the presence of two positively charged residues identified in this motif is congruent with its interaction with DNA.

In summary, *FcNAC1* is implicated as a transcriptional regulator during the softening process of the Chilean strawberry fruit. The evidence provided will contribute to understanding the regulatory network that takes place during development and ripening of *F*. *chiloensis* fruit. Future work to identify or generate mutants of *FcNAC1* will aim to confirm the level of this involvement.

## Materials and Methods

### Plant material

Chilean strawberry fruit at different stages of development (C1, C2, C3 and C4) and vegetative tissues (flowers, roots, leaves, runners and stem) were collected from plants grown in a commercial field at Contulmo city, Biobío Region, Chile (latitude 38°04′8.6′′S, longitude 73°14′2.96′′W). The fruit was classified into four different developmental stages according to weight and color of the receptacle and achenes as previously reported: C1, small fruit with green receptacle and green achenes; C2, large fruit with green receptacle and red achenes; C3 turning stage, fruit of white receptacle and red achenes; and C4, ripe fruit with pink receptacle and red achenes^5^. After harvest, the peduncle and calyx of each fruit were removed, and the fruit cut longitudinally into two halves, frozen in liquid nitrogen and stored at −80 °C for later use.

### Isolation and cloning of strawberry FcNAC1 cDNA

From an SSH (suppressive substractive hybridization) library prepared form *F*. *chiloensis* fruit at different developmental stages^30^ an EST contig was identified as differentially expressed, and by means of BLAST analysis (https://blast.ncbi.nlm.nih.gov/Blast.cgi) its tentative homology to plant NAC sequences was revealed.

With the aim to isolate the full-length cDNA sequence of FcNAC1, RACE (Rapid Amplification of cDNA Ends) reactions using RNA from ripe fruit stage as template were performed using the BD Smart RACE cDNA Amplification kit (Clontech, USA). Two 3′-RACE PCR reactions were performed using the Universal Primer A Mix (Clontech) and primers FcNAC1-RACE1 and FcNAC1-RACE2 (Table 1). The expected amplicons obtained were cloned into pGEM-T Easy Vector (Promega, USA) and sequenced at Macrogen, Inc. (Korea). Then a new set of primers was designed to obtain the full-length cDNA of FcNAC1: FcNAC1-Full-length-F and FcNAC1-Full-length-R (Table 1); the CACC sequence was incorporated to the forward primer for directional cloning. This amplified sequence was cloned into the pENTER^TM^/SD/D-TOPO^®^ vector included in the pENTER^TM^ Directional TOPO^®^ Cloning kit (Invitrogen). PCR reactions were employed to confirm the presence and directionality of FcNAC1 sequence by using the primers mentioned before. Then plasmids were purified employing GeneJET Plasmid Miniprep kit (Thermo Scientific^TM^), and sequenced at Macrogen, Inc. (Korea). The amino acid sequence of FcNAC1 has been submitted to NCBI under the accession number: KP966107.1.

### Transient expression of FcNAC1 in Nicotiana benthamiana leaves and sub-cellular localization

Gateway^®^ LR Clonase^TM^ II Enzyme Mix kit (Invitrogen) was used to perform a 35S:*FcNAC1*-*GFP* construction. The manufacturer’s instructions were used for recombination using equivalent amounts of entry vector (160 ng/ul) and destination vector (pK7WGF2.0).

The *35 S:FcNAC1-GFP* construct cloned in pK7FWG2.0 vector was introduced into *Agrobacterium tumefaciens* strain LBA4404 ElectroMAX^TM^ (Invitrogen) by thermic shock in liquid nitrogen. Transformed bacteria were plated on a selective medium yeast mold agar containing streptomycin (PhytoTechnology Laboratories) and spectinomycin (PhytoTechnol. Lab.) at a final concentration of 100 µg/ml each. Resistant colonies were analyzed by PCR for the presence of full-length *FcNAC1* gene using the primers mentioned before. A positive colony was cultured in selective YM (100 ml) and incubated at 28 °C until an O.D._600_ between 0.6 and 0.8. Agroinfiltration suspension was used to inject the abaxial face of young tobacco leaves (two weeks old) and samples were analyzed after three days of infiltration. Syto^®^ 84 Orange Fluorescent Nucleic Acid stain (Thermo Scientific^TM^) was used to label the nucleus. Subcellular localization of FcNAC1 in transient transformed leaves samples was analyzed through visualization of the tissue under a confocal fluorescence microscope (Carl Zeiss Confocal microscopy LMS 700) employing phase contrast image.

### RNA extraction and Expression analysis by Real Time PCR (RT-qPCR)

Samples of RNA were extracted from 2 g of *F*. *chiloensis* fruit bulk from each developmental stage (C1, C2, C3 and C4) and other vegetative tissues (flowers, roots, stem, runners and leaves) using the CTAB method with modifications^50^. RNA samples were treated with DNase I amplification grade (Invitrogen), and then cleaned using an RNeasy Plant Mini Kit (Qiagen). cDNA synthesis using First Strand cDNA Synthesis Kit (Fermentas) was performed following the manufacturer’s instructions. Three biological sample replicates from each fruit stage or tissue were considered. Specific primers designed for 5′-UTR region of *FcNAC1* and 3′-UTR region of glyceraldehyde 3-phosphate dehydrogenase (*FcGAPDH1*; as internal control)^51^ were used (Table 1). Primers for 5′-UTR region of *FcNAC1* were designed by Primer3 (http://frodo.wi.mit.edu/primer3/). Primers were tested by RT-PCR.

For RT-qPCR analysis Maxima SYBR Green/ROX qPCR Master Mix (2×) (Fermentas) was used following manual instructions, in a DNA engine Opticon 2 Real-Time PCR System (MJ Research, Watertown, MA). The amplification efficiency was determined through a dilution series of a cDNA mix prepared from different fruit samples. All qPCR reactions were carried on in duplicates, and water was used as negative controls in each run. Relative expression levels correspond to means of three biological replicates ± standard deviation normalized against the gene expression level of *FcGAPDH1* (constitutive gene), employing C1 stage as the calibrator of fruit samples and leaves as the calibrator of vegetative tissue samples. Different letters of significance indicate significant differences in expression level (*p* ≤ 0.05).

### Genome walker and analysis of FcNAC1 promoter sequence

The *FcNAC1* promoter sequence was obtained by the Genome Walker technique using GenomeWalker^TM^ Universal kit (Clontech) according to the manufacturer’s instructions using specific primers designed (Table 1). DNA samples were extracted from 2 g of *F*. *chiloensis* leaves bulk using the CTAB method with modifications^50^. DNA samples were subjected to enzymatic digestions with different restriction enzymes: EcoRI, XbaI, HindIII and XhoI, following the protocol described in the kit. After cloning, sequencing and assembling these sequences, the TATA box and cis-elements were predicted using the online sequence analysis program PlantCARE (http://bioinformatics.psb.ugent.be/webtools/plantcare/html/)^52^.

### Phylogenetic analysis

The deduced amino acid sequences were analyzed using the translate tool of ExPASy (http://ca.expasu.org). The similarity search was performed using the local alignment tool (BLAST, National Center for Biotechnology Information, USA). The multiple alignments of amino acid sequences were performed using ESPript 3.0 (http://espript.ibcp.fr/ESPript/ESPript/)^53^ incorporating secondary structural information. The phylogenetic tree was built using MEGA software (Version 5.2, http://megasoftware.net)^54^, using neighbor joining method and 1000 bootstrap replicates.

### Hormonal treatments

*Fragaria chiloensis* fruit at the C2 stage were treated with 1 mM ABA (Abscisic acid) and 1 mM NAA (1-Naphthaleneacetic acid) according to Opazo *et al*.^51^ with some modifications. For these assays, C2 maturation stage was considered as it has been reported that the cross-talk between AUX and ABA occurs at this stage of fruit development in *Fragaria*^34^. Fruit lots of 24 fruits were incubated during 10 min with ABA, ANA or remained untreated. Samples of 6 fruits were collected just after treatment (10 min), and after 1 h, 2 h and 12 h of treatment; the fruit was immediately frozen in liquid nitrogen and stored at −80 °C until use. RNA extractions and RT-qPCR reactions were performed as indicated above.

### Dual luciferase assay of transiently transformed Nicotiana benthamiana leaves

Two DNA fragments corresponding to promoter sequences of genes related to cell wall remodeling, *PL* (*Pectate lyase*, GenBank KC527025; 1038 bp) and *EXP2* (*Expansin 2*, GenBank KC527027; 847 bp), which contain cis*-*elements in to response NAC transcription factors, were isolated by the introduction of restriction sites: 5′ NotI and 3′ NcoI for *PL*, and 5′ BamHI and 3′ NotI for *EXP2*. Then these sequences were inserted into the cloning site of pGreenII 0800-*LUC* vector^33^. The promoter sequence of *FvDFR* (Dihydroflavonol reductase (AB211139); 1560 bp) was used as a positive control of the technique. The internal control was *Renilla* (*REN*) *LUC* driven by 35 S promoter. The full-length coding sequence of *FcNAC1* (GenBank AKC96459.1) was inserted into the pHEX2 vector under the 35 S promoter by Gateway, according to the manufacturer’s instructions. *FvMYB10* (GenBank EU155163.1) and *FvBHLH* (Basic helix loop helix, XP_004308377; 1932 bp) fused to pHEX2 vector were used as positive control of the technique. Since *GUS* gene (*uidA*) has not been related to cell wall remodeling pathways, it was fused to pHEX2 vector to function as a negative control. All the constructs were transformed into *A*. *tumefaciens* GV3101, and these cultures were incubated at 28 °C for 2 days. Leaf disks were punched 3 days after infiltration, and then subjected to luminescence assay. Luc/Ren ratio activity was measured using the Orion Microplate Luminometer (Berthold Detection Systems, http://www.titertek-berthold.com/) as previously reported Espley *et al*.^55^ and reported as mean of four technical replicates ± standard deviation.

### Homology modeling of NAC domain of FcNAC1

The search for an appropriate crystal structure available in the Protein Data Bank (PDB)^56^ with the closest homology to FcNAC1 was performed through BLAST. The structure of ANAC019 (PDB: 3SWM chain A) was selected as template to build the NAC domain present in FcNAC1. To build the structure the pipeline described by Morales-Quintana *et al*.^57^ was used. The molecular dynamics was performed following the strategy reported by Morales-Quintana *et al*.^58^.

### Statistical analysis

For gene expression analysis a random design with three biological replicates and two technical replicates were used. Statistical analyses were performed using the SPSS v.14 package. Analysis of variance (ANOVA) and significant differences were determined at *p* ≤ 0.05 (LSD Fisher test).

## Electronic supplementary material

Supplementary figures and tables

## References

[1] Bringhurst RS (1990). Cytogenetics and evolution in American Fragaria. Hort Sci..

[2] Hancock JF, Lavín A, Retamales JB (1999). Our southern strawberry heritage: *Fragaria chiloensis* of Chile. Hort Sci..

[3] Qin Y, da Silva T (2008). J. A, Zhang, L. & Zhang, S. Transgenic strawberry: state of the art for improved traits. Biotech Adv..

[4] Retamales JB, Caligari PDS, Carrasco B, Guillermo S (2005). Current status of the Chilean native strawberry and the research needs to convert the species into a commercial crop. Hort. Sci..

[5] Figueroa CR (2008). Softening rate of the Chilean strawberry (*Fragaria chiloensis*) fruit reflects the expression of polygalacturonase and pectate lyase genes. Postharvest Biol. Technol..

[6] Prasanna V, Prabha TN, Tharanathan RN (2007). Fruit ripening phenomena–an overview. Cri. Rev. F. Sci. and Nutrit..

[7] Brummell DA (2006). Cell wall disassembly in ripening fruit. Funct. Plant Biol..

[8] Vicente AR, Saladié M, Rose JKC, Labavith JM (2007). The linkage between cell wall metabolism and fruit softening: looking to the future. J. Sci. Food Agric..

[9] Figueroa CR (2010). Changes in cell wall polysaccharides and cell wall degrading enzymes during ripening of *Fragaria chiloensis* and *Fragaria x ananassa* fruits. Sci. Hort..

[10] De Oliveira TM (2011). Analysis of the NAC transcription factor gene family in citrus reveals a novel member involved in multiple abiotic stress responses. TGG..

[11] Shan W (2012). Molecular characterization of banana NAC transcription factors and their interactions with ethylene signalling component EIL during fruit ripening. J. Exp. Bot..

[12] Zhu M (2013). A new tomato NAC (NAM/ATAF1/2/CUC2) transcription factor, SlNAC4, functions as a positive regulator of fruit ripening and carotenoid accumulation. Plant Cell Physiol..

[13] Zhou H (2015). Molecular genetics of blood-fleshed peach reveals activation of anthocyanin biosynthesis by NAC transcription factors. Plant J..

[14] Souer E, Van Houwelingen A, Klos D, Mol J, Koes R (1996). The no apical meristem gene of Petunia is required for pattern formation in embryos and flowers and is expressed at meristem and primordia boundaries. Cell.

[15] Aida M, Ishida T, Fukaki H, Fujisawa H, Tasaka M (1997). Genes involved in organ separation in Arabidopsis: an analysis of the cup-shaped cotyledon mutant. Plant Cell.

[16] Delessert C (2005). The transcription factor ATAF2 represses the expression of pathogenesis-related genes in Arabidopsis. Plant J..

[17] Wang X (2009). The Arabidopsis ATAF1, a NAC transcription factor, is a negative regulator of defense responses against necrotrophic fungal and bacterial pathogens. Mol. Plant-Microbe Interact..

[18] Hibara K, Takada S, Tasaka M (2003). CUC1 gene activates the expression of SAM-related genes to induce adventitious shoot formation. Plant J..

[19] Kou XH (2014). Molecular Characterization and expression analysis of NAC family transcription factors in tomato. Plant Mol. Biol..

[20] Rushton PJ (2008). Tobacco transcription factors: novel insights into transcriptional regulation in the Solanaceae. Plant Physiol..

[21] Hu R (2010). Comprehensive analysis of NAC domain transcription factor gene family in Populus trichocarpa. BMC Plant Biol..

[22] Nuruzzaman M (2010). Genome-wide analysis of NAC transcription factor family in rice. Gene.

[23] Le DT (2011). Genome-wide survey and expression analysis of the plant-specific NAC transcription factor family in soybean during development and dehydration stress. DNA Res..

[24] Puranik S, Sahu PP, Srivastava PS, Prasad M (2012). NAC proteins: regulation and role in stress tolerance. Trends Plant Sci..

[25] Kim SG, Kim SY, Park CM (2007). A membrane-associated NAC transcription factor regulates salt-responsive flowering via FLOWERING LOCUS T in Arabidopsis. Planta.

[26] Zhu T, Nevo E, Sun D, Peng J (2012). Phylogenetic analyses unravel the evolutionary history of NAC proteins in plants. Evolution..

[27] Ma N (2014). Overexpression of tomato SlNAC1 transcription factor alters fruit pigmentation and softening. BMC Plant Biol..

[28] Nieuwenhuizen NJ (2015). Natural variation in monoterpene synthesis in kiwifruit: transcriptional regulation of terpene synthases by NAC and EIN3-like transcription factors. Plant Physiol..

[29] Zhong R, Lee C, Ye ZH (2010). Evolutionary conservation of the transcriptional network regulating secondary cell wall biosynthesis. Trends Plant Sci..

[30] Pimentel P, Salvatierra A, Moya-león MA, Herrera R (2010). Isolation of genes differentially expressed during development and ripening of *Fragaria chiloensis* fruit by suppression subtractive hybridization. J. Plant Physiol..

[31] Liu YZ (2009). Identification and expression pattern of a novel NAM, ATAF, and CUC-like gene from Citrus sinensis Osbeck. Plant Mol. Biol. Rep..

[32] Nakano Y (2015). NAC-MYB based transcriptional regulation of secondary cell wall biosynthesis in land plants. Frontiers in. Plant Sci..

[33] Concha CM (2013). Methyl jasmonate treatment induces changes in fruit ripening by modifying the expression of several ripening genes in *Fragaria chiloensis* fruit. Plant Physiol. Biochem..

[34] Hellens RP (2005). Transient expression vectors for functional genomics, quantification of promoter activity and RNA silencing in plants. Plant Meth..

[35] Ooka H (2003). Comprehensive analysis of NAC family genes in *Oryza sativa* and *Arabidopsis thaliana*. DNA Res..

[36] He XJ, Mu RL, Cao WH (2005). AtNAC2 a transcription factor downstream of ethylene and auxin signal pathways is involved in salt stress response and lateral root development. Plant J..

[37] Wang H, Zhao Q, Chen F, Wang M, Dixon RA (2011). NAC domain function and transcriptional control of a secondary cell wall master switch. Plant J..

[38] Han Q (2012). Identification and expression pattern of one stress-responsive NAC gene from Solanum lycopersicum. Mol. Biol. Rep..

[39] Hussey SG (2011). *SND2*, a NAC transcription factor gene, regulates genes involved in secondary cell Wall development in *Arabidopsis* fibres and increases fibre cell area in *Eucalyptus*. BMC Plant. Biol..

[40] Zhong R, Zheng-Hua Y (2014). Complexity of the transcriptional network controlling secondary Wall biosynthesis. Plant Sci..

[41] Opazo MC (2010). Characterization of two divergent cDNAs encoding xyloglucan endotransglycosylase/hydrolase (XTH) expressed in *Fragaria chiloensis* fruit. Plant Sci..

[42] Kang C (2013). Genome-scale transcriptomic insights into early-stage fruit development in woodland strawberry *Fragaria vesca*. Plant Cell..

[43] Symons GM (2012). Hormonal changes during non-climacteric ripening in strawberry. J. Exp. Bot..

[44] Jia HF (2011). Abscisic acid plays an important role in the regulation of strawberry fruit ripening. Plant Physiol..

[45] Zhu M (2014). Molecular characterization of six tissue-specific or stress-inducible genes of NAC transcription factor family in tomato (*Solanum lycopersicum*). J. Plant Growth. Regul..

[46] Nuruzzaman M, Sharoni AM, Kikuchi S (2013). Roles of NAC transcription factors in the regulation of biotic and abiotic stress responses in plants. Front. Microbiol..

[47] McCarthy RL, Zhong R, Ye ZH (2011). Secondary wall NAC binding element (SNBE), a key cis-acting element required for target gene activation by secondary wall NAC master switches. Plant Signal. Behav..

[48] Olsen AN, Ernst HA, Lo Leggio L (2005). NAC transcription factors: structurally distinct functionally diverse. Plant Sci..

[49] Welner DH (2012). DNA binding by the plant-specific NAC transcription factors in crystal and solution: a firm link to WRKY and GCM transcription factors. Biochem. J..

[50] Chang S, Puryear J, Cairney J (1993). A simple and efficient method for isolating RNA from pine trees. Plant Mol. Biol. Rep..

[51] Opazo MC, Lizana R, Pimentel P, Herrera R, Moya-León MA (2013). Changes in the mRNA abundance of *FcXTH1* and *FcXTH2* promoted by hormonal treatments of *Fragaria chiloensis* fruit. Postharvest Biol. Technol..

[52] Lescot M (2002). PlantCARE: a database of plant *cis*-acting regulatory elements and a portal to tools *in silico* analysis of promoter sequences. Nucl. Acid. Res..

[53] Robert X, Gouet P (2014). Deciphering key features in protein structures with the new ENDscript server. Nucl. Acid. Res..

[54] Tamura K, Dudley J, Nei M, Kumar S (2004). Integrative software for Molecular Evolutionary Genetics Analysis and sequence alignment. Brief. Bioinform..

[55] Espley RV (2009). Multiple repeats of a promoter segment causes transcription factor autoregulation in red apples. Plant Cell.

[56] Rose PW (2011). The RCSB Protein Data Bank: redesigned web site and web services. Nucl. Acid. Res..

[57] Morales-Quintana L, Fuentes L, Gaete-Eastman C, Herrera R, Moya-León MA (2011). Structural characterization and substrate specificity of VpAAT1 protein related to ester biosynthesis in mountain papaya fruit. J. of Mol. Graph. and Mod..

[58] Morales-Quintana L, Nuñez-Tobar MX, Moya-León MA, Herrera R (2013). Molecular dynamics simulation and site-directed mutagenesis of alcohol acyltransferase: a proposed mechanism of catalysis. J. of Chem. Infor. and Mod..

