# Supplementary material for: Characterization of a ripening-related transcription factor FcNAC1 from Fragaria chiloensis fruit
Source: Sci Rep. 2018 Jul 12;8:10524. doi: 10.1038/s41598-018-28226-y (PMC6043618; doi:10.1038/s41598-018-28226-y)
Supplement: Supplementary file 1 — Supplementary figures and tables [file 41598_2018_28226_MOESM1_ESM.pdf]

Characterization of a ripening-related transcription factor *FcNAC1* from *Fragaria chiloensis* fruit.

CARRASCO-ORELLANA, C., STAPPUNG, Y., MENDEZ-YAÑEZ, A., ALLAN, A.C., ESPLEY, R.V., PLUNKETT, B. J., MOYA-LEON, M. A. and HERRERA, R*

Supplementary Figures.

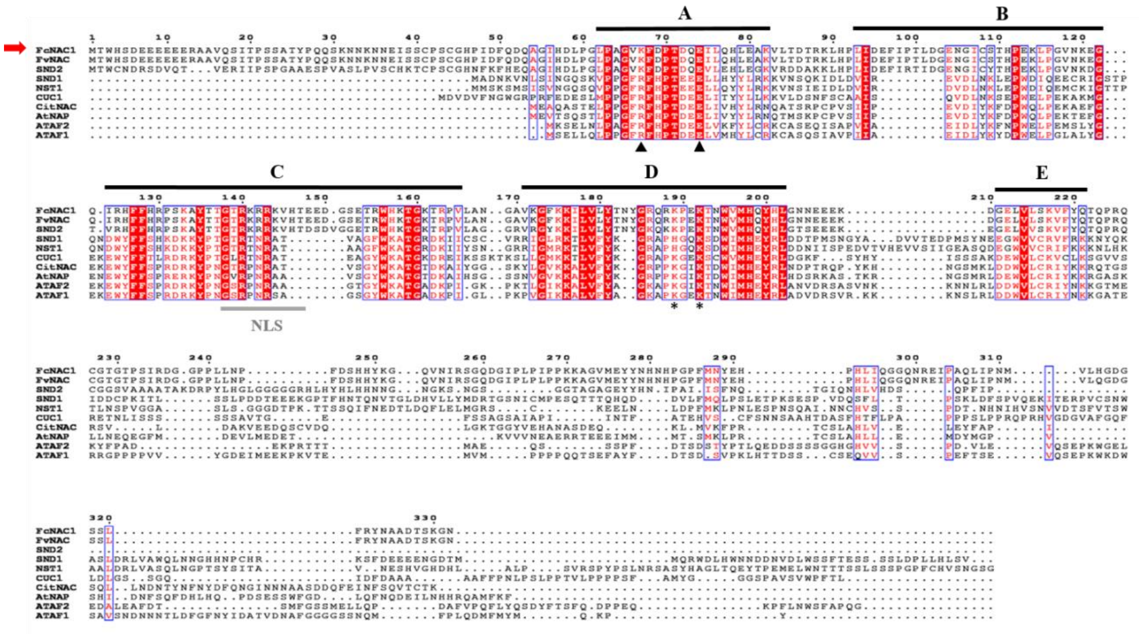

Supplementary figure 1. Multiple alignment of FcNAC1 (marked with red arrow) sequence and other NAC family members where the conserved amino acids are marked with white letters and similar residues with red letters. Important residues for homodimerization are marked with triangles and those important for the DNA interaction with asterisks. The nuclear localization signal prediction is labeled in grey color. The alignment of sequences was performed with ESPript3.0 software (http://espript.ibcp.fr/ESPript/ESPript/). The accession numbers of NAC members are: ATAF1 (NM_100054.3), ATAF2 (NM_147856.4), AtNAP (NM_105616.4), CitNAC (EF185419.1), CUC1 (EU550396.1), NST1 (NM_130243), SND1 (EF101892), and SND2 (NC_003075.7); FvNAC (XP_004293895) related to FcNAC1 (AKC96459). The NLS label indicates the nuclear localization signal.

FcNAC1

Predicted NLSs in query sequence

```
MTWHSDEEEEEERAAVQSITPSSATYPQQSKNNKNNEISSCPSCGHPIDF 50
QDQAGIHDLPGLPAGVKFDPTDQEILQHLEAKVLTDTRKLHPLIDEFIPT 100
LDGENGICSTHPEKLPGVNKEGQIRHFFHRPSKAYTTGTRKRRKVHTEED 150
GSETRWHKTGKTRPVLANGAVKGFKKILVLYTNYGRQRKPEKTNWVMHQY 200
HLGNNEEEKDGELVLSKVFYQTQPRQCGTGTPSIRDGGPPLLNPFDSHHY 250
KGQVNIRSGQDGIPLPIPPKKAGVMEYYNHNHPGPFMNYEHPHLIQGGQN 300
REIPAQLIPNMVLHGDGSSLFRYNAADTSKGN                   332
```

Predicted monopartite NLS		
Pos.	Sequence	Score
138	GTRKRRKVHTE	9.5
138	GTRKRRKVHT	10

Supplementary figure 2. Nuclear localization signal prediction of FcNAC1 amino acid sequence by NLS Mapper software.

A

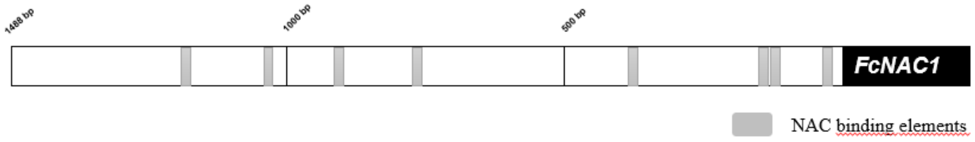

B

Position	Strand	Similar score	Hit Sequence
1124	+	1	tacACGCAag
1042	-	0.92	atTACGTgga
209	-	0.86	gctACGTAcg
221	+	0.87	ggTACGTata
221	-	0.88	ggtACGTAta
452	+	0.91	aaTACGTatc
452	-	0.92	aatACGTAtc
778	+	0.86	cgTACGTatg
778	-	0.86	cgtACGTAtg
11	-	0.99	gtTGCGTggc
1042	+	0.97	atTACGTg
837	-	1	ggTTGACct
12	+	0.99	ttGCGTGgct

C

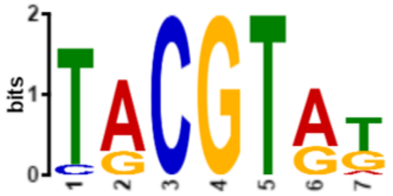

Supplementary figure 3. Promoter sequence of the *FcNAC1* gene and *in-silico* analysis of cis-elements related to NAC TF**.** A) Representation of FcNAC1 promoter sequence, which was analyzed with the Plant Pan 2.0 database. In color are represented cis-elements of response to interaction with NAC transcription factors. B) Table with the result of the analysis of Plant Pan 2.0, where is possible appreciate the different elements of response to NAC, its sequence and positioning. C) Representative logo of cis elements of the NACBS type made with MEME software.

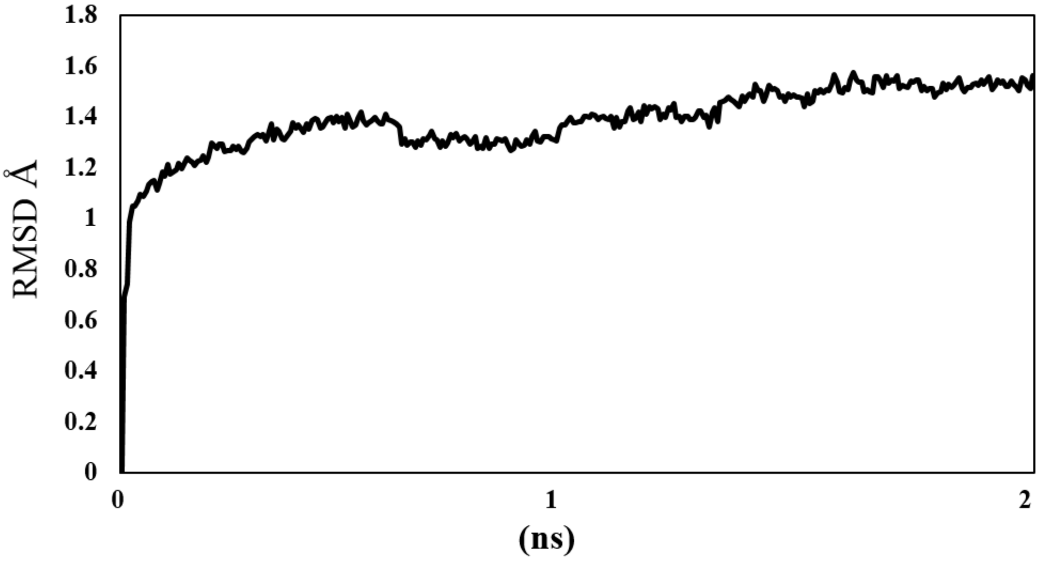

Supplementary figure 4. The MD simulation curves of FcNAC1 domain**.** The RMSD plot calculated for backbone atoms of FcNAC1 monomer domain during its MD simulation (2 nano seconds).

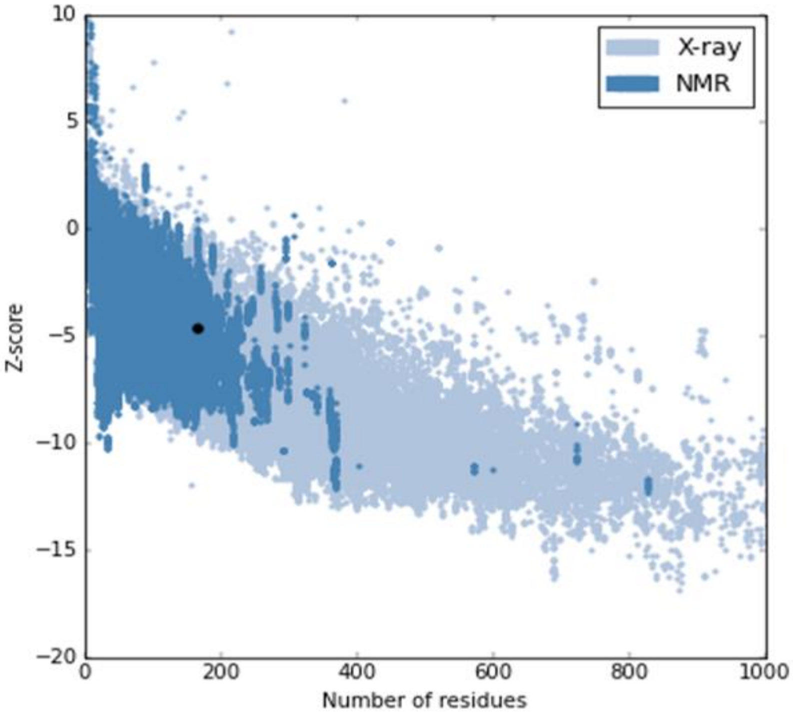

Supplementary figure 5. Evaluation of the FcNAC1 monomer domain by Prosa software.

Supplementary material

Supplementary Table 1. Sequence identity (%) between complete NAC sequences by ClustalO.

	FcNAC1	FvNAC	SND2	SND1	NST2	NST1	TIP	NAC1	CUC1	PeNAM	CUC2	SNAC1	ATAF2	SlNAC1	ATAF1	OsNAC6	HvNAC6	NAM-B1	AtNAC2	PvNAP	CitNAC	AtNAP
FcNAC1	100	99.4	62.63	21.1	24.58	22.54	22.98	21.1	25.32	26.5	28.8	24.38	25.95	25.85	28.06	26.21	27.49	23.75	23.5	23.22	24.64	22.69
FvNAC		100	62.63	21.94	25	22.95	23.4	21.52	25.32	26.92	29.2	24.38	25.95	26.34	28.06	26.21	27.49	24.14	23.5	23.7	24.64	23.61
SND2			100	20.8	22.49	21.66	23.92	21.9	23.47	24.88	25	25.13	26.52	25.13	27.72	26.8	26.53	25.31	24.88	21.72	24.39	22.17
SND1				100	50.96	54.12	34.4	33.95	35.36	38.34	34.44	34.16	36.07	32.64	34.33	33.2	34.32	31.34	30.18	39.82	35.44	38.2
NST2					100	65.87	34.66	34.46	34.52	38.96	34.38	33.63	35.85	32.47	32.31	33.9	34.62	31.6	30.71	39.17	37.5	38.29
NST1						100	36.5	34.63	34.94	39.69	36.16	32.23	36.61	33.33	34.45	34.41	33.88	31.85	29.07	39.47	37.23	39.24
TIP							100	29.5	36.61	34.02	32.46	30.63	33.59	32	34.6	32.36	33.57	28.26	29.87	35.44	38.72	38.27
NAC1								100	41.11	41.97	42.19	35.56	37.5	34.15	34.31	32.13	32.95	31.16	34.64	37.83	38.74	33.62
CUC1									100	57.82	53.04	39.83	40.91	36.96	38.7	37.97	38.52	36.3	37.17	40.61	41.18	38.46
PeNAM										100	60.31	36.07	44.69	38.93	39.75	39.92	39.36	37.38	36.26	42.92	41.74	40.93
CUC2											100	35.09	37.92	36.02	37.6	35.79	37.04	35.05	32.17	38.43	41.87	37.85
SNAC1												100	53.57	53.08	51.75	53.23	55.6	43.88	46.59	48.36	50	47.27
ATAF2													100	62.41	60.84	58.27	60.69	46.83	47.27	53.2	50.5	48.24
SlNAC1														100	64.62	61.19	63.14	44.12	45.02	47.49	50	46.64
ATAF1															100	66.67	65.31	45.38	45.8	51.66	50.24	49.77
OsNAC6																100	82.29	44.65	45.76	46.9	49.33	46.49
HvNAC6																	100	45.93	45.93	46.35	48.67	46.35
NAM-B1																		100	49.55	48.21	49.03	46.74
AtNAC2																			100	54.2	49.59	48.41
PvNAP																				100	61.57	61.2
CitNAC																					100	62.4
AtNAP																						100

Supplementary Table 2. Sequence identity (%) among NAC domains determined by ClustalO.

	FcNAC1	FvNAC	SND2	TIP	NST2	SND1	NST1	NAC1	CUC1	PeNAM	CUC2	SNAC1	ATAF2	SlNAC1	ATAF1	OsNAC6	HvNAC6	CitNAC	PvNAP	AtNAP	NAM-B1	AtNAC2
FcNAC1	100	100	84.29	30.6	29.84	29.03	31.45	29.6	34.68	37.1	37.9	33.06	31.71	33.33	33.33	32.52	32.52	33.06	30.65	30.65	35.48	33.87
FvNAC		100	84.29	30	29.84	29.03	31.45	29.6	34.68	37.1	37.9	33.06	31.71	33.33	33.33	32.52	32.52	33.06	30.65	30.65	35.48	33.87
SND2			100	30	29.03	29.84	29.84	28	33.87	35.48	36.29	36.29	33.33	34.96	35.77	34.96	35.77	33.06	30.65	31.45	37.9	35.48
TIP				100	51.97	55.91	54.33	49.6	57.94	57.94	57.14	53.6	57.26	54.84	55.65	55.65	54.84	57.6	57.6	56.8	53.17	51.59
NST2					100	83.59	89.84	58.4	59.2	60	57.6	51.2	54.84	50.81	51.61	50.81	52.42	55.2	55.2	53.6	52.8	51.2
SND1						100	90.62	55.2	61.6	62.4	60.8	54.4	54.84	52.42	54.03	51.61	51.61	57.6	58.4	57.6	54.4	53.6
NST1							100	58.4	63.2	64	61.6	53.6	54.84	51.61	52.42	51.61	51.61	56.8	57.6	56	53.6	52.8
NAC1								100	62.4	64	64	55.2	54.03	54.03	53.23	54.03	54.03	53.6	54.4	50.4	52	52.8
CUC1									100	89.76	85.83	57.6	60.48	58.06	59.68	58.06	58.06	62.4	63.2	60.8	62.2	59.84
PeNAM										100	90.55	57.6	62.1	60.48	63.71	62.1	59.68	64	66.4	62.4	65.35	62.2
CUC2											100	59.2	62.1	61.29	65.32	64.52	62.1	63.2	63.2	60.8	62.2	62.2
SNAC1												100	75.81	83.06	77.42	78.23	76.61	68.8	65.6	65.6	73.6	76
ATAF2													100	87.1	84.68	83.06	81.45	67.74	66.94	65.32	71.77	70.16
SlNAC1														100	86.29	87.1	85.48	70.97	65.32	66.94	73.39	72.58
ATAF1															100	90.32	87.9	69.35	68.55	66.94	74.19	75
OsNAC6																100	94.35	70.16	68.55	68.55	73.39	75
HvNAC6																	100	70.16	66.13	66.13	73.39	73.39
CitNAC																		100	88	88.8	76.8	76
PvNAP																			100	88.8	73.6	75.2
AtNAP																				100	73.6	74.4
NAM-B1																					100	86.61
AtNAC2																						100
